# SIRT1 deacetylated and stabilized XRCC1 to promote chemoresistance in lung cancer

**DOI:** 10.1038/s41419-019-1592-3

**Published:** 2019-05-01

**Authors:** Neelum Aziz Yousafzai, Qiyin Zhou, Wenxia Xu, Qiqi Shi, Jinye Xu, Lifeng Feng, Hui Chen, Vivian Yvonne Shin, Hongchuan Jin, Xian Wang

**Affiliations:** 1 Department of Medical Oncology, Key Laboratory of Biotherapy of Zhejiang Province, Sir Run Run Shaw Hospital, Medical School of Zhejiang University, Hangzhou, China; 2 Laboratory of cancer Biology, Key Laboratory of Biotherapy of Zhejiang Province, Sir Run Run Shaw Hospital, Medical School of Zhejiang University, Hangzhou, China; 3 Department of Pathology, Sir Run Run Shaw Hospital, Medical School of Zhejiang University, Hangzhou, China; 4 Department of Surgery, The University of Hong Kong, Hong Kong SAR, Hong Kong, China

**Keywords:** Cancer, Lung cancer

## Abstract

Chemoresistance is one of the most important challenges in the clinical management of lung cancer. SIRT1 is a NAD dependent protein deacetylase and implicated in diverse cellular processes such as DNA damage repair, and cancer progression. SIRT1 is upregulated in chemoresistant lung cancer cells, genetic knockdown or chemical inhibition of SIRT1 reversed chemoresistance by enhancing DNA damage and apoptosis activation, accompanied with XRCC1 degradation. E3 ligase β-TrCP catalyzed the poly-ubiquitination of XRCC1 to promote its proteasome-dependent degradation. SIRT1 bound and deacetylated XRCC1 at lysine K260, K298 and K431, preventing it from β-TrCP-dependent ubiquitination. Mutations of these three lysine sites in XRCC1 abrogated the interaction with β-TrCP and prolonged the half-life of XRCC1 protein. Here, we describes SIRT1 confers chemoresistance to lung cancer cells by deacetylating and stabilizing XRCC1. Therefore, targeting SIRT1 might be a new strategy to manage the chemoresistance of lung cancer, and probably other cancers.

## Introduction

Lung cancer is most death responsible disease in both men and women worldwide. Treatment of lung cancer remains a big challenge, although great developments such as EGFRTKI (tyrosine kinase inhibitor) based targeted therapy have been achieved^1^. However, lung cancer cells are able to become resistant to many drugs due to genetic and epigenetic alterations^2^. Over the past decades, platinum-based chemotherapy is the most standard choice for the treatment of various solid cancers including lung cancer^3^. The mechanisms underlying resistance to platinum-based chemotherapy has been extensively explored to provide rational strategies for overcoming chemoresistance. SIRT1 (sirtuin1) which belongs to the class III HDACs (histone deacetylases) family have drawn emerging diverse functions like silent information regulator, genome stability, longevity in response to metabolic and other stress conditions^4,5^. For example, SIRT1 overexpression enhanced resistance to paclitaxel and cisplatin in endometria carcinoma cell lines^6^. SIRT1 overexpression reduced apoptosis and promotes DNA damage repair by activating multiple repair pathways like homologous recombination (HR), nucleotide excision repair (NER), base excision repair (BER) and non-homologous end joining (NHEJ)^7^, as all these pathways has been regulated by SIRT1 through deacetylation including Nijmegen breakage syndrome protein (NBS1),^8^ Ku70^9^ and apurinic/apyrimidinic endonuclease^10^. Meanwhile, SIRT1 was able to deacetylate histones H1, H3, and H4 to remodel chromatin conformations^11^. As a result, SIRT1 is also upregulated in various cancers, including melanoma, colon, prostrate, breast, liver, lymphoma, leukemia and sarcomas^12–15^. However, the function and relevance of SIRT1 to chemoresistance of lung cancer cells was largely unknown.

In present study, we found that SIRT1 expression was upregulated in chemotherapeutic resistant lung cancer cells. It interacted with and stabilized X-ray cross-complementing-1 (XRCC1) by disrupting the acetylation-dependent interaction of XRCC1 with β-TrCP E3 ligase.

Suppression of SIRT1 by siRNAs and SIRT1 inhibitors promoted XRCC1 degradation and restored chemosensitivity.

## Materials and Methods

### Reagents and antibodies

DMEM, RPMI-1640 (Invitrogen, Carlsbad, CA, USA), EX527, Nicotinamide, SRT1720 (Selleckchem, Shanghai, China), Cisplatin, ADM, VP-16, Cycloheximide, MG132 (Sigma Aldrich, Shanghai, China), Puromycin (Life Technologies/Gibco), Trizol reagent (Invitrogen), anti-XRCC1, anti-Ku80, anti-SIRT1, anti- γ-H2AX, anti-c-PARP1 were purchased from Abcam, Shanghai, China, anti-Cullin 1, anti-β-TrCP, anti- c-Caspase3 were purchased from Cell Signaling Technology, Shanghai, China, anti-α-Tubulin, anti-Flag, anti-poly Ubiquitin, anti-Pan acetyl lysine, anti-HA, anti-HA agarose beads, anti-Flag beads, GST- Sepharase beads, were purchased from Sigma Aldrich, Shanghai, China.

### Cell culture

Human lung cancer cell line H460 and human embryonic kidney cell line HEK293 was purchased from the Type Culture Collection of the Chinese Academy of Sciences (Shanghai, China), H460 cells were cultured in RPMI-1640 and HEK293 in DMEM medium supplemented with 10% of FBS (fetal bovine serum). The cells were maintained at 37 ℃_ in a 5% CO_2_ humidified incubator. Chemoresistant cells H460-R were developed from the parental cell line H460 subjected to determined gradient exposure of cisplatin, adriamycin and VP-16 for about 12 months, through increasing various concentration of chemotherapy from 0.05 μg/ml to 2 μg/ml, the cells acquired resistance.

### Cell viability assay

H460 and H460-R cells were seeded at a density of 7000 cells per well in 96 well plates. Briefly, after 12–16 h. cells were treated with various concentration of above mentioned drugs for different time interval 24, 48, 72 h. The cell viability was determined using MTS reagents according to the manufacturer’s instructions.

### Plasmids and siRNAs transfections

For cell transfection, lentivirus SIRT1 plasmid was purchased from GeneChem Company (Shanghai, China). H460 cells were transfected with lentivirus vector carrying SIRT1 according to the manufacturer’s instructions. Cells were stably expressed by selection with puromycin 50 μg/ml (Life Technologies/Gibco). Flag-XRCC1, Flag- β-TrCP, HA-UB, and GFP-XRCC1 plasmids were constructed by GeneChem Company (Shanghai, China) as described previously^16,17^. Cells were seeded in 6-well plates for overnight, 2 μg of plasmids were mixed with X-treme GENE HP DNA Transfection Reagent (Roche Applied Science). For siRNAs transfection cells were transfected with specific SIRT1 and β-TrCP siRNA with Lipofectamine TM RNAiMAX transfection reagent (Thermofisher Scientific) according to the manufacturer’s instructions^18^. All these siRNAs were constructed by GenePharma (Shanghai, China) and the sequence detail are in Supplementary Table 1.

### Flow cytometry analysis

Cells were treated with SIRT1 genetic, chemical inhibitors, SIRT1 activator and chemotherapeutic drugs, treated cells were washed with cold PBS. Apoptotic cell was determined using the FITC Annexin V Apoptosis Detection Kit I (BD Bioscience, Bedford, MA, USA) following to the manufacturer’s instructions. Trypsinized ells were washed twice with cold PBS and then mixed well in 1 × Binding Buffer. FITC 5 μl of Annexin V and 5 μl of PI was added in cellular suspensions, and then incubated at room temperature for 15 min in dark place. All apoptotic cells were analyzed by Flow cytometry.

### Immunofluorescence assay

Cells were grown on the slides chamber and treated with SIRT1 genetic and chemical inhibitors, activator and chemotherapeutic drug cisplatin are mentioned above. Treated cells were washed with PBS and following protocol performed as previously reported^19^. Cells were incubated with anti γ-H2AX antibody rabbit monoclonal with Alexa Fluor 488-conjugated rabbit antibody respectively. DAPI mounted cells were analyzed by LSM 710 confocal microscope, images of cells were consecutively obtained using Zeiss AIM software.

### RNA extraction and Real-time quantitative PCR (qRT-PCR)

Total RNA was extracted from cells using Trizol reagent (Invitrogen) according to the manufacturer’s instructions and concentration of RNA was quantified by NanoDrop 2000 (Nanodrop, Wilmington, Del., USA). Reverse transcription (RT)-PCR was performed using high quality cDNA Reverse Transcription Kit (Thermofisher Scientific Inc., Shanghai, China). Briefly, the mRNA expression level was determined by qRT-PCR (ABI 7500 Real-Time PCR Applied Biosystems, CA USA) using CYBR Green Master Mix (Roche, Shanghai, China) with 2 μg total RNA. All primers sequences are listed in Supplementary Table 2.

### Immunoprecipitation and Western blotting

For Immunoprecipitation assay, cells were washed with cold PBS. Briefly, cells were suspended in cold NP40 lysis buffer or RIPA buffer (Beyotime, Jiangsu, China). Total protein or lysates was quantified by BCA protein kit method (Bio-Rad Laboratories, Hercules, CA, USA). Lysates were incubated at 4 °C with Flag coated beads or specific antibody overnight followed by addition of agarose beads for 2–4 h. respectively. The precipitant complexes were dissolved in 2x lysis buffer and resolved by SDA-PAGE. Western blot analysis was performed as described previously^20^, SDS-PAGE were transferred to PVDF membranes and incubated with appropriate primary antibodies overnight at 4 °C, then washed and incubated at room temperature with specific secondary antibody. The membranes were exposed with enhanced chemiluminescence (EMD Millipore, Billerica, MA, USA) in Amersham Imager 600 system (GE Healthcare Life Sciences, Shanghai, China).

### Mass spectrometry analysis

HEK293 cells were transfected with Flag-XRCC1 and β-TrCP, and stably expressed, cell lysates were immunoprecipitated and subjected to affinity purification with anti-Flag antibody. The immunoprecipitated complex was resolved by SDS-PAGE and stained with coomassie blue, the specific size bands were retrieved and analyzed by Mass spectrometry liquid chromatography (MS/LC).

### Site directed mutagenesis

To investigate the role of mutation, The QuikChange II Site-Directed Mutagenesis Kit (Applied Biosystem, CA.USA) was followed to generate XRCC1 and β-TrCP site-directed mutation according to the manufacturer’s instructions. Briefly, PCR was performed to create mutagenesis, WT of XRCC1 and β-TrCP plasmid was used as template. Mutant and WT primers were used to amplify the plasmid by PCR, then transformed it into DH5α. All mutant samples were confirmed by sequencing and primers sequence is listed in Supplementary Table 2.

### Glutathione-S-Transferase (GST) pull-down

For protein–protein interactions, various length primers of XRCC1 were synthesized by GenePharma (Shanghai, China). Sequence of GST pull-down primers are listed in Supplementary Table 2. DNA fragments of XRCC1 were amplified by PCR and cloned in EcoR1 and Not1 sites of pGEX-4T-1 vector, GST-XRCC1 fusion protein was expressed in *E.* *coli*, purified and interacts with GSH-sepharase 4B beads, four independent GST–XRCC1 pull-downs and two GST-control pull-downs were performed as described previously^21^ (ThermoFisher, SCIENTIFIC, USA) according to the manufacturer’s instructions. Briefly, precleared cell lysates were incubated with GSH-sepharase beads at 4 °C overnight. Conjugated beads complex were washed with cold washing buffer, dissolved in 2 × lysis buffer and resolved by SDS-PAGE. Gel was stained with coomassie blue and observed specific bands when compared with protein ladder. Next, HEK293 cells were transfected with Flag-β-TrCP, cells were lysed using NP-40 lysis buffer, and co-immunoprecipitated with GST-XRCC1 and GST control beads, protein–protein interaction was analyzed by immunoblotting.

### Statistical analysis

Data are expressed as the mean ± S.D. The significance of the data between experimental groups was performed by the Student’s t tests. P values less than 0.05 were considered to be statistically significant.

## Results

### SIRT1 expression is increased to promote chemoresistance

**Fig. 1:**
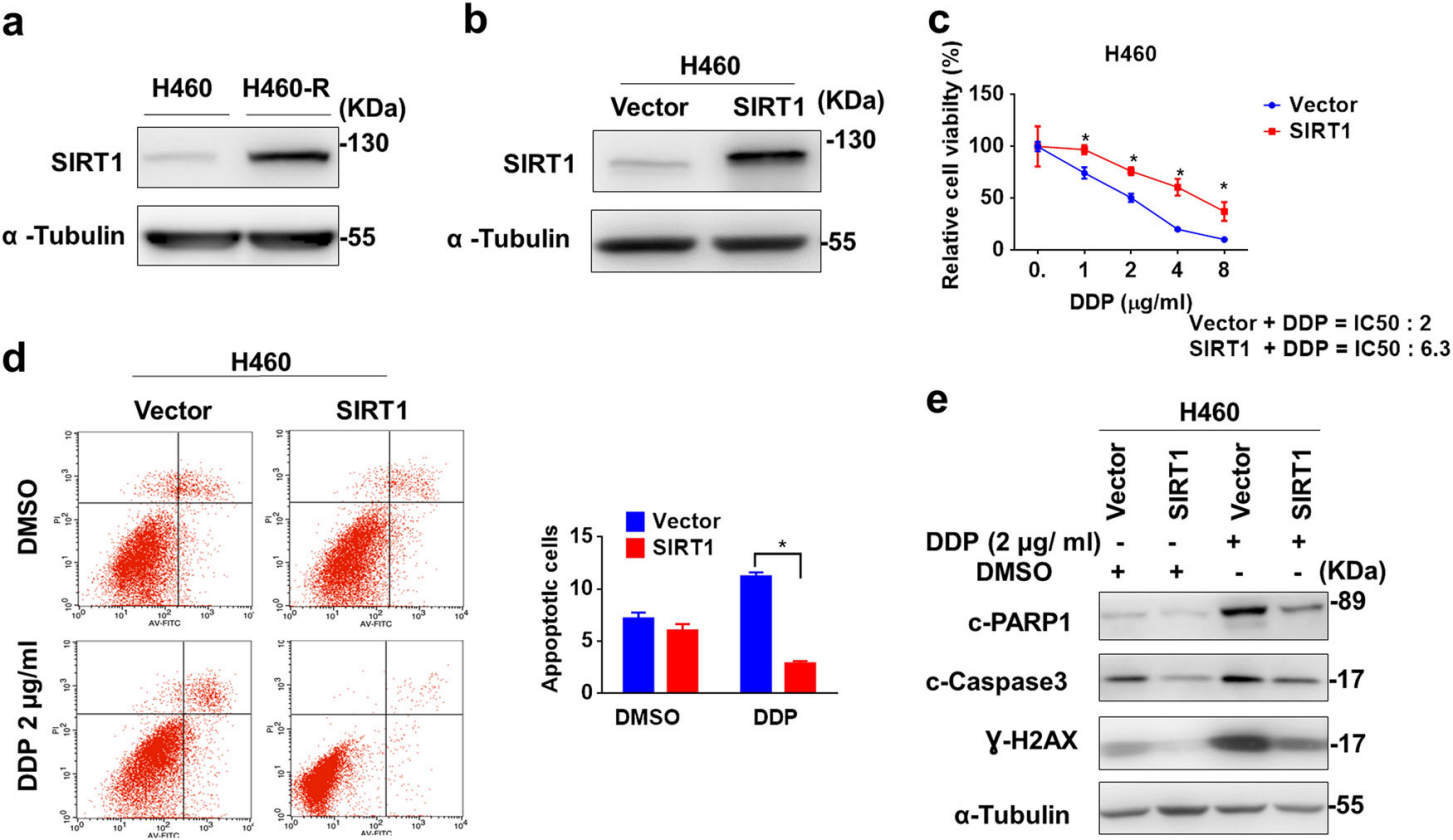
SIRT1 expression is increased to promote chemoresistance. **a** SIRT1 expression in H460-R and H460 lung cancer cells were determined by Western blotting. **b** Western blot analysis of SIRT1 expression in H460 cells transfected with lentivirus carrying empty vector or SIRT1-expressing vector. **c** The viability of H460 cells with SIRT1 overexpression after treatment with cisplatin was indicated were measured by MTS assay. **d** Analysis of apoptosis by Annexin V/propidium Iodide staining with Flow cytometry. Data represents in triplicate and shown as the mean ± S.D. *p *<* 0.05. **e** Protein level of cleaved-PARP1, cleaved-Caspase3 and Ɣ-H2AX in H460 cells after cisplatin treatment were analyzed by Western blotting

We first investigated SIRT1 expression in H460 cells and chemoresistant H460-R (DDP, ADM, and VP-16) cells. As shown in Fig. 1a, SIRT1 protein expression was notably upregulated in H460-R. To explore the relevance of SIRT1 upregulation to chemoresistance, we transfected H460 cells with SIRT1-expression vector carrying lentivirus to increase SIRT1 expression (Fig. 1b). As a result, H460 became more resistant to chemotherapeutic drug cisplatin significantly (Fig. 1c, IC50 increased from 2 μg/ml to 6.3 μg/ml 72 h. after SIRT1 overexpression), accompanied with less apoptosis revealed by Annexin V/PI staining (Fig. 1d) and Western blotting-based caspase activity analysis (Fig. 1e).

### Knockdown of SIRT1 reverses chemoresistance

**Fig. 2:**
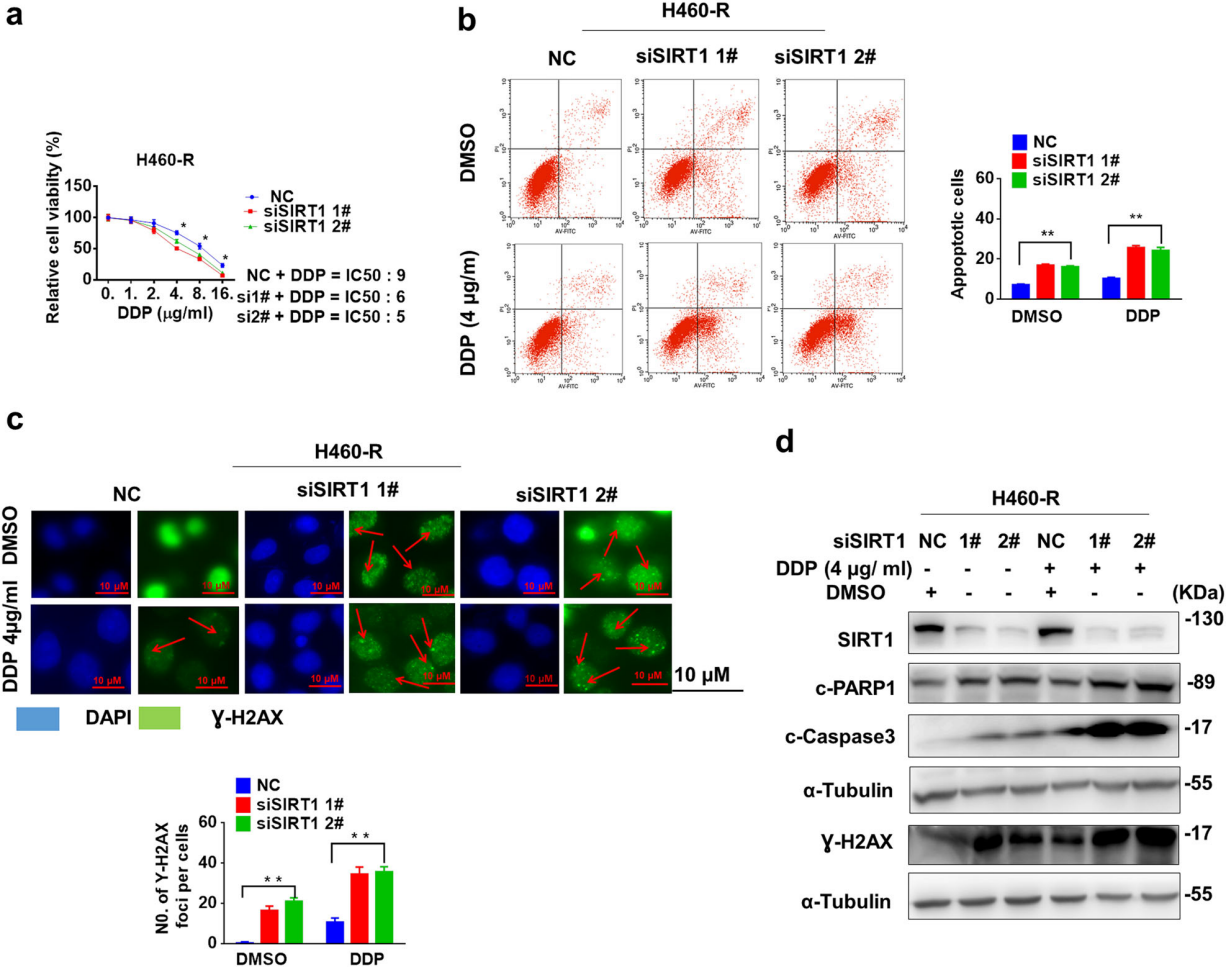
Knockdown of SIRT1 reverses chemoresistance. **a** The viability of SIRT1-depleting H460-R cells after cisplatin treatment were measured by MTS assay. **b** and **c** Apoptosis of SIRT1-depleting H460-R cells after cisplatin treatment and Ɣ-H2AX levels were analyzed by Flow cytometry (**b**) and fluorescent microscope (**c**). Data represents in triplicate and shown as the mean ± S.D. *p *<* 0.05. **d** Protein level of cleaved-PARP1, cleaved-Caspase 3 and Ɣ-H2AX in H460-R cells after cisplatin treatment were analyzed by western blotting

To further study the relevance of SIRT1 upregulation to chemoresistance, we knocked down SIRT1 expression in H460-R cells with small interfering RNAs (siRNAs). SIRT1 knockdown significantly reduced cell viability (Fig. 2a), and increased sensitivity of H460-R cells to cisplatin and induced significant apoptosis activity in knockdown cells with combination of cisplatin (Fig. 2b). More Ɣ-H2AX positive foci were observed in cisplatin-treated cells after SIRT1 knockdown (Fig. 2c), indicating of severer DNA damage. Consequently, more apoptosis was activated in cisplatin-treated cells after SIRT1 knockdown (Fig. 2d).

### Chemical modulation of SIRT1 activity affects chemosensitivity to DDP

**Fig. 3:**
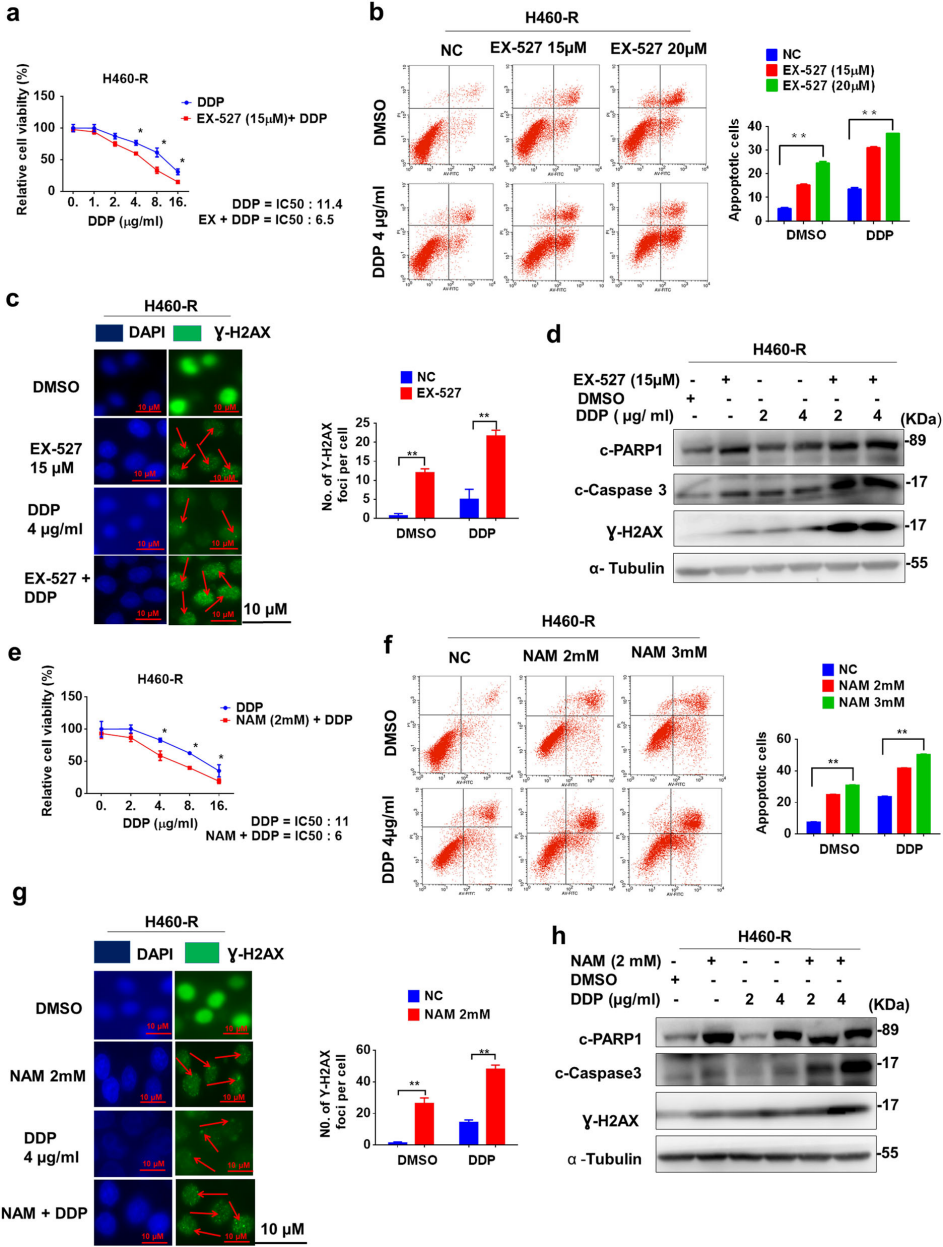
Chemical inhibitors of SIRT1 impair chemoresistance. **a** Cisplatin-induced viability inhibition of H460-R with or without EX-527 treatment (72 h.) were measured by MTS assay. **b**–**d** Apoptosis and Ɣ-H2AX (DNA damage) levels in H460-R with or without EX-527 treatment (48 h.) were analyzed as in Fig. 2b–d, respectively. **e**–**h** Cisplatin-induced viability inhibition and apoptosis activation of H460-R with or without NAM treatment (48 h.) were measured as in **b**–**d**, respectively

We next analyzed the effect of SIRT1 inhibition by chemical inhibitors on chemoresistance of lung cancer cells. Two independent inhibitors EX-527 and nicotinamide (NAM) were used. After EX-527 treatment, the chemosensitivity of H460-R cells to cisplatin was increased (Fig. 3a, IC50 was decreased from 11.4 to 6.5 μg/ml). More apoptosis and DNA damage were induced by cisplatin in the presence of SIRT1 inhibition by EX-527 (Fig. 3b–d). Similarly, NAM also potentiated cisplatin-induced viability inhibition (Fig. 3e), and apoptosis activation (Fig. 3f–h). Furthermore, we also investigated the effect of SIRT1 inhibitors on sensitivity of H460 cells to other drugs such as adriamycin (ADM). As shown in Supplementary Fig. 1, chemosensitivity of H460-R cells to ADM was also increased after SIRT1 inhibition. In contrast, SIRT1 activator SRT1720 conferred chemoresistance to H460 cells (Supplementary Fig. 2). Upon SRT1720 treatment, the IC50 of H460 cells to DDP was increased from 2 to 6 μg/ml (Supplementary Figure 2a). Consistently, DDP-induced apoptosis and Ɣ-H2AX foci were reduced in H460 cells treated with SRT1720 (Supplementary Figure 2b–d).

### SIRT1 upregulates XRCC1 protein through inhibiting its degradation

**Fig. 4:**
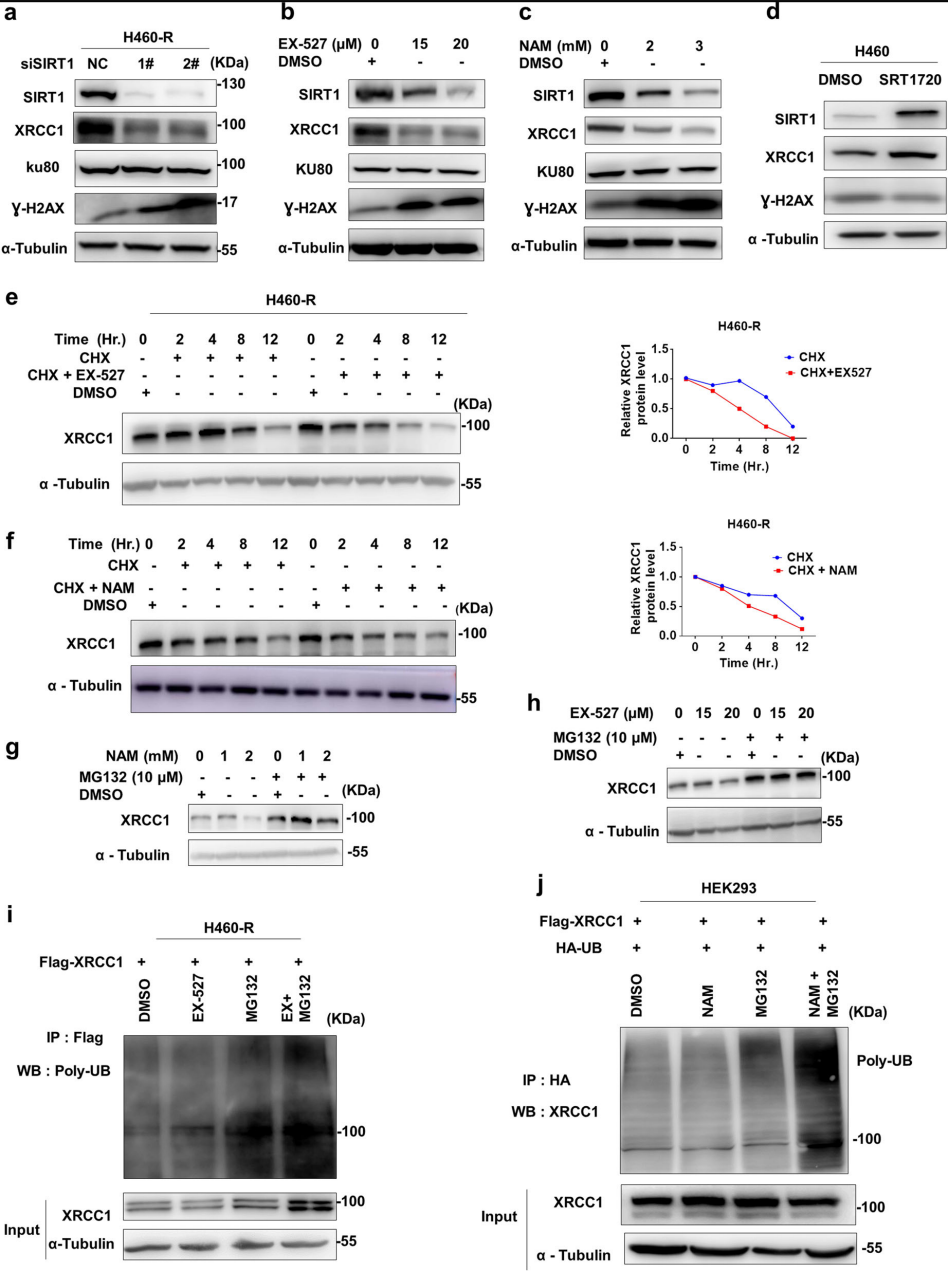
SIRT1 upregulates XRCC1 protein through inhibiting its degradation. **a** Protein expressions in H460-R cells before and after SIRT1 knockdown were analyzed by western blotting. **b**, **c** Protein expressions in H460-R cells before and after EX-527 (**b**) or NAM (**c**) treatment were analyzed by western blotting. **d** Protein expressions in H460 cells before and after SIRT1 overexpression were analyzed by western blotting. **e**, **f** XRCC1 protein levels in EX-527 (15 μM) (**e**) or NAM (2 mM) -treated (**f**). H460-R cells after cycloheximide (CHX, 50 μg/ml) treatment were determined by western blotting. **g**, **h** XRCC1 protein levels in EX- 527 (**g**) or NAM**-**treated (**h**) H460-R cells in the presence of MG132 treatment for 6 h. were analyzed by Western blotting. **i**, **j** H460-R cells expressing Flag- XRCC1 and HEK-293 HA tagged Ubiquitin were treated with EX-527, NAM and MG132. Ubiquitinated proteins were immunoprecipitated (IP) with anti-HA antibody and anti-Flag antibody for Western blotting with anti-XRCC1 antibody

Given the effect of enhanced DNA damage upon SIRT1 inhibition, we explored the effect of SIRT1 inhibition on the expression of proteins involved in DNA damage repair. Together with the increase of Ɣ-H2AX, XRCC1 but not KU70 was also increased after genetic (Fig. 4a) or chemical inhibition (Fig. 4b and c) of SIRT1. On the other hand, XRCC1 was upregulated and Ɣ-H2AX was downregulated in H460 cells after XRCC1 overexpression (Fig. 4d). In the presence of SIRT1 inhibition (EX-527 in Fig. 4e and NAM in Fig. 4f), the half-life of XRCC1 protein was shortened significantly, indicating that SIRT1 might inhibit XRCC1 protein degradation. The effect of chemical and genetic inhibitors on XRCC1 mRNA level was not significant, as shown in Supplementary Fig. 3a–c. Indeed, proteasome inhibitor MG132 succeeded to rescue both EX-527 and NAM induced XRCC1 downregulation (Fig. 4g, h), accompanied with increased ubiquitination of XRCC1 (Fig. 4i, j). Collectively, these results suggested that SIRT1 stabilized XRRC1 by inhibiting its ubiquitination-dependent degradation.

### β –TrCP is the E3 ligase for XRCC1

**Fig. 5:**
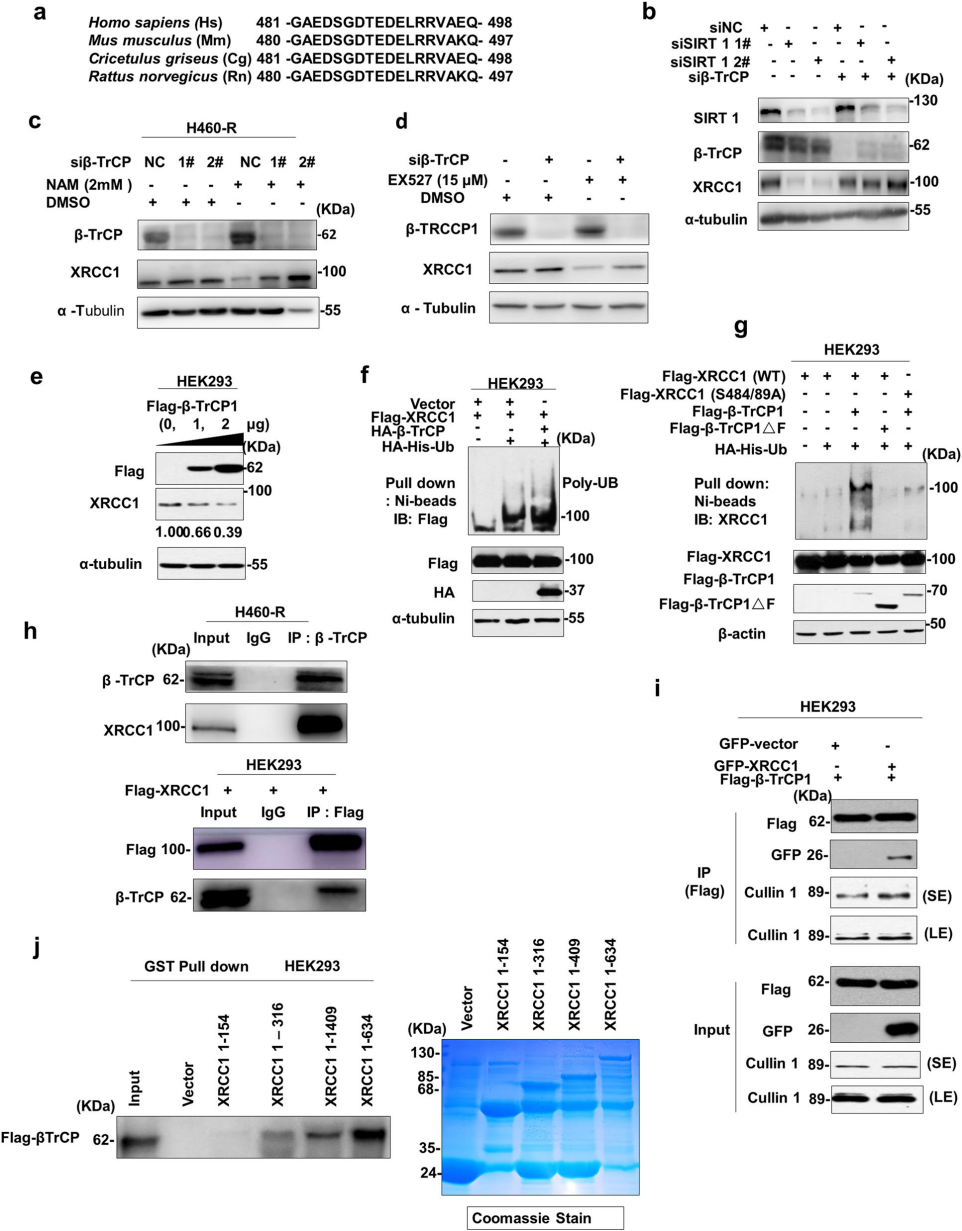
β –TrCP is the E3 ligase for XRCC1. **a** Alignment of the candidate degron sequence in XRCC1 for β-TrCP binding. **b–d** Expression of XRCC1 in H460-R cells transfected with SIRT1 and/or β-TrCP siRNA and SIRT1 inhibitors were analyzed by Western blot. **e** Immunoblot (IB) analysis of whole cell lysate (WCL) and immunoprecipitates (IP) from HEK293 transfected with Flag- β-TrCP and detected low XRCC1 interactions with high expression of β-TrCP. **f**, **g** Ubiquitination analysis of WCL were transfected with WT and mutant Flag-β-TrCP and Flag- XRCC1 with HA-ubiquitin vector, nickel beads were used to pull-down ubiquitin protein. **h**, **i** Interaction of XRCC1 with β-TrCP in both endogenous and exogenous cells was determined by co-immunoprecipitation (Co-IP). **j** Interaction of Flag- β-TrCP with GST-XRCC1 was analyzed by GST-pull-down assay stained with coomassie blue and Western blot

To further investigate the molecular mechanism of SIRT1-inhibited XRCC1 degradation, we tried to screen the potential E3 ligase responsible for XRCC1 ubiquitination. Interestingly, we found a conserved potential DSGXX degron motif for β-TrCP recognition in XRCC1 proteins from various species (Fig. 5a), indicating that XRCC1 ubiquitination might be catalyzed by β–TrCP. To support this assumption, knockdown of β-TrCP was indeed able to rescue XRCC1 downregulation induced by genetic (Fig. 5b) or chemical (Fig. 5c, d) inhibition of SIRT1. Moreover, exogenous β-TrCP induced XRCC1 downregulation dose-dependently (Fig. 5e) and promoted XRCC1 ubiquitination (Fig. 5f). However, XRCC1 protein carrying mutations in phosphodegron failed to be ubiquitinated by β-TrCP (Fig. 5g). Enzyme activity defective mutant of β-TrCP was also not able to ubiquitinate XRCC1 (Fig. 5g), confirming that β-TrCP is the bona fide E3 ligase responsible for XRCC1 ubiquitination. Both endogenous (Fig. 5h) and exogenous (Fig. 5i) XRCC1 can form a complex with β-TrCP in vivo. Specifically, the N terminus (1–154) of XRCC1 seemed to be dispensable for its interaction with β-TrCP (Fig. 5j).

### SIRT1 deacetylates XRCC1 to impairs its interaction with β-TrCP

**Fig. 6:**
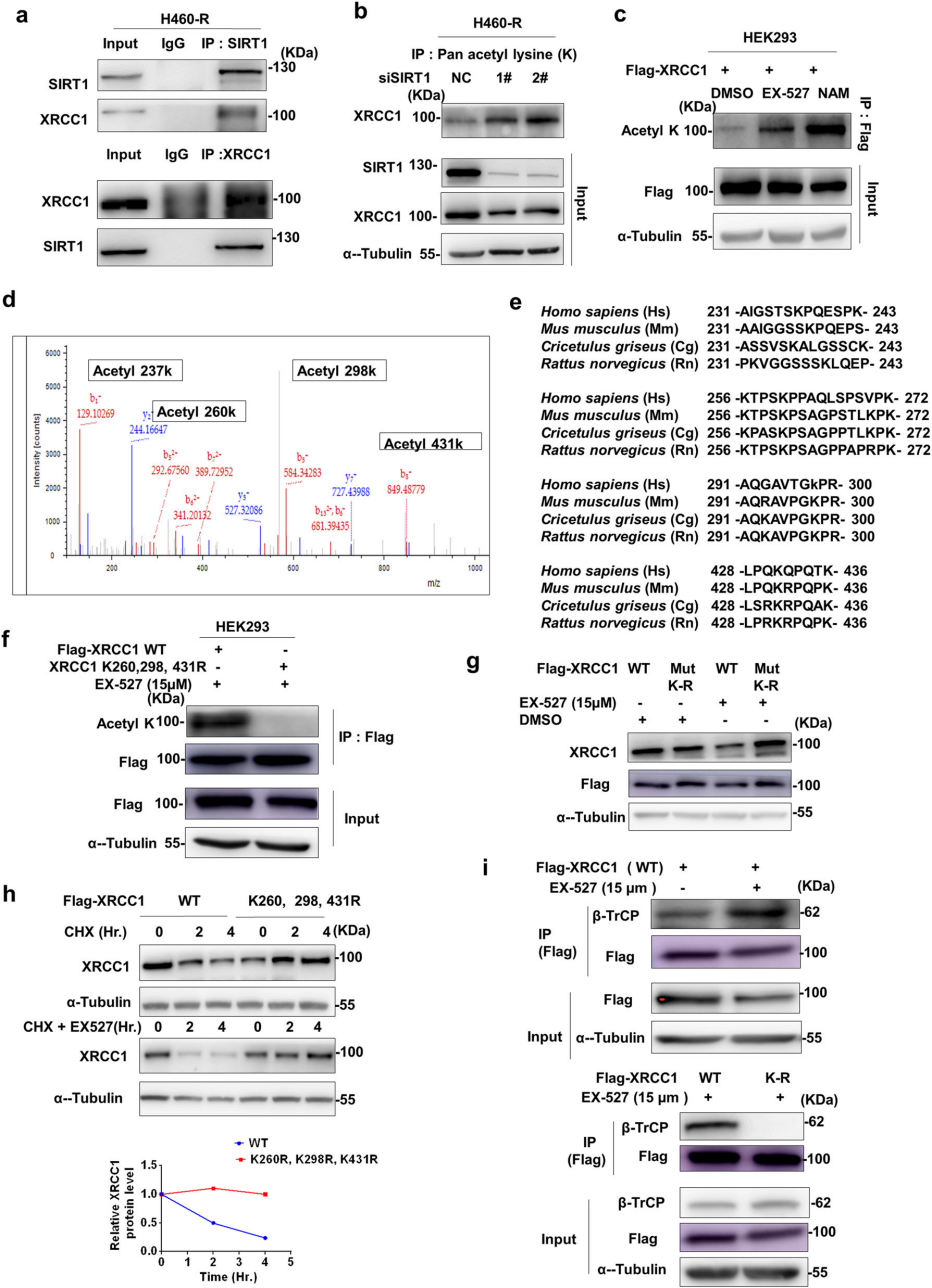
SIRT1 deacetylates XRCC1 to impair its interaction with β-TrCP. **a** Interaction of SIRT1 with XRCC1 in H460-R cells was determined by co-immunoprecipitation (Co-IP). **b** Effect of SIRT1 siRNAs on acetylation of XRCC1 in H460-R cells was determined by anti- XRCC1 antibody immunoblotting after immunoprecipitation with anti-Pan-acetyl lysine antibody. **c** Effect of SIRT1 inhibitors on acetylation of XRCC1 in H460-R cells was determined as in (**b**). **d** The acetylation of XRCC1 in NAM-treated H460-R cells was analyzed by mass spectrometry (MS). The MS spectrum of peptide XRCC1 showed that lysine 237, 260, 298, 431 was acetylated in NAM-treated XRCC1. **e** Alignment of XRCC1 acetylated sites in various species. **f** Acetylation of wild type or mutated XRCC1 in HEK293 cells was determined by anti- Pan-acetyl lysine antibody immunoblotting after immunoprecipitation with anti-flag antibody. **g** Effect of EX-527 on the expression of wild type or mutated XRCC1 in HEK293 cells was determined by anti-flag immunoblotting. **h** Effect of EX-527 on half-life of wild-type or mutated XRCC1 in CHX-treated HEK293 cells was analyzed by immunoblotting with anti-flag antibody. **i** Interaction of wild type or mutated XRCC1 with β-TrCP in HEK293 cells before and after treatment with EX-527 were analyzed by co-immunoprecipitation with anti-Flag antibody and immunoblotting with anti- β-TrCP antibody

Next, we explored how inhibition of SIRT1 promoted XRCC1 degradation. Firstly, we found that endogenous SIRT1 could interact with XRCC1 in vivo (Fig. 6a), indicating that SIRT1 might affect the interaction of XRCC1 with β-TrCP through regulating the lysine acetylation of XRCC1. As expected, the lysine acetylation of XRCC1 was increased after genetic (Fig. 6b) or chemical (Fig. 6c) inhibition of SIRT1, similar to p53 acetylation (K382) which was well-known to be regulated by SIRT1 (Supplementary Figure 4a and b). Four potentially acetylated lysine (K237, K260, K298 and K431), were identified by Mass spectrometry liquid chromatography (MS/LC) (Fig. 6d). To further endorse lysine acetylation of XRCC1, three conserved lysine residues except K237 were changed into arginine by site-directed mutagenesis (Fig. 6e). The lysine acetylation was completely lost in such a mutant (Fig. 6f), confirming the identification results of Mass spectrometry. Moreover, EX-527 failed to downregulate the expression of XRCC1 K-R mutant (Fig. 6g). The half-life of this mutant was also significantly extended (Fig. 6h). Importantly, β- TrCP could interact with wild-type XRCC1 but not this K-R mutant (Fig. 6i). Therefore, SIRT1 deacetylates XRCC1 to impair its interaction with β-TrCP and subsequent degradation.

## Discussion

Lung cancer is the one of the highest leading cause of deaths worldwide^22^. Its treatment is challenging due to acquired or intrinsic drug resistance mainly resulting from genome instability. Recently, targeted therapy such as tyrosine kinase inhibitors (TKIs) for EGFR (epithelial growth factor receptor) has improved the clinical management of lung cancer. However, novel mutations leading to TKI-resistance are merging and chemotherapy remains the major adjuvant treatment approach for lung cancer. Therefore, there is an urgent need to overcome chemoresistance. In the present study, we found that SIRT1 is overexpressed to promote chemoresistance in lung cancer. By deacetylating proteins involved in critical processes such as apoptosis in response to various stresses, SIRT1 was assumed to play an important role in promoting oncogenesis^23^. Previous studies have shown that SIRT1 was overexpressed in several cancers like breast cancer, prostate tumors^24^, ovarian epithelial tumors^25^, soft tissue sarcomas^26^ and cutaneous T-cell lymphomas^27^. Therefore, targeting SIRT1 might be a new therapy option for chemoresistant lung cancer, and probably other cancers.

Many chemotherapeutic drugs such as cisplatin function to induce DNA damages. As a result, chemoresistant cancer cells will evolve to acquire potency in DNA damage repair capacity. DNA repair can be regulated in a protein-acetylation-dependent manner. Protein acetylation regulators such as SIRT1 and p300 are regulated by TSPYL2 nucleosome protein in response to DNA damage^28^. Moreover, sinomenine induces apoptosis in malignant glioma cells and p53 acetylation through downregulation of SIRT1^29^. In this report, we found that XRCC1 was stabilized to promote chemoresistance in lung cancer. It was involved in efficient repair of DNA single-strand breaks formed by exposure to ionizing radiation and alkylating agents and essential for microhomology-mediated end joining (MMEJ) repair of double strand breaks^30^. Previously, we have demonstrated an important role of XRCC1 in repairing DNA damage and contributing to chemoresistance in gastric cancer^19,31^. KDM5B (lysine demethylase 5B) was upregulated in chemoresistant gastric cancer cells to demethylate H3K4 and enable XRCC1 recruitment to DNA damage lesions for efficient DNA damage repair^19^. On the other hand, XRCC1 was activated by casein kinase 2 (CK2) and degraded via ubiquitin-proteasome pathway in a TXNL1 (thioredoxin-like protein 1)-dependent manner^31^. However, the regulation of XRCC1 ubiquitination remains unknown. Herein, we found that β-TrCP is the bona fide E3 ligase for the ubiquitination of XRCC1. Interestingly, knockdown of β-TrCP rescued XRCC1 from ubiquitination-dependent degradation induced by SIRT1 inhibition and SIRT1 inhibition promotes strong interaction between β-TrCP- XRCC1. Acetylation-defective mutant of XRCC1 failed to interact with β-TrCP, escaped from ubiquitination-dependent degradation and became a more stable protein. SIRT1 deacetylates XRCC1, thus impairing its ubiquitination by β-TrCP. Generally, β-TRCP E3 ligase requires a phosphodegron to recognize its target for Ubiquitination and degradation of β-catenin 17. Recent study has been reported that that MLN4924, a small molecule inhibitor of neddylation modification, it induces mitochondrial fission-to-fusion conversion in breast cancer cells through suppressing degradation of fusion-promoting protein mitofusin1 (MFN1) and ubiquitylation by SCFβ-TrCP E3 ligase^32^.

Our results indicated that other post-translational modifications such as lysine acetylation are emerging as new signals for β-TrCP probably other E3 ligases as well to recognize their targets. Indeed, lysine acetylation of glutamine synthetase (GS) triggered recognition by the CRL4CRBN E3 ubiquitin ligase, resulting in its ubiquitylation and degradation in response to high glutamine concentrations^33^. Similarly, p300 mediated acetylation of MDM2 E3 ligase on Lys182 and Lys185 and stabilize MDM2 in numerous cancers. Therefore, acetyldegron was proposed to function as phosphodegrons for the substrates recognition of ubiquitin ligases^34^. This would be valuable to understand the regulation of ubiquitination-dependent protein degradation by identification of more interactions of E3 ligases with substrates under various circumstances. Certainly, more investigations including the identification of particular acetyltransferases and the dynamic regulation of acetylation under different conditions are warranted.

In summary, our study exposed that both genetic and chemical inhibition of SIRT1 reversed chemoresistance by enhancing DNA damage and apoptosis activation. Lysine acetylation of XRCC1 by SIRT1 inhibition protects it from β-TrCP-dependent Ubiquitination-proteasomal degradation, thus restoring chemosensitivity. Therefore, targeting SIRT1 might be a new strategy to manage the chemoresistance of lung cancer, and probably other cancers.

## Supplementary information

Supplementary Figures and Tables

## References

[1] Yonesaka K (2018). An HER3-targeting antibody-drug conjugate incorporating a DNA topoisomerase I inhibitor U3-1402 conquers EGFR tyrosine kinase inhibitor-resistant NSCLC. Oncogene.

[2] Gottesman MM, Fojo T, Bates SE (2002). Multidrug resistance in cancer: role of ATPdependent transporters. Nat. Rev.Cancer.

[3] Zappa C, Mousa SA (2016). Non-small cell lung cancer: current treatment and future advances. Transl. Lung Cancer Res..

[4] Longo VD, Kennedy BK (2006). Sirtuins in aging and age-related disease. Cell.

[5] Frye RA (1999). Characterization of five human cDNAs with homology to the yeast SIR2 gene: Sir2-like proteins (sirtuins) metabolize NAD and may have protein ADP ribosyltransferase activity. Biochem. Biophys. Res. Commun..

[6] Asaka R, Miyamoto T (2015). Sirtuin 1 promotes the growth and cisplatin resistance of endometrial carcinoma cells: a novel therapeutic target. Lab. Invest..

[7] Majidinia Maryam, Sadeghpour Alireza, Mehrzadi Saeed, Reiter Russel J., Khatami Nasrin, Yousefi Bahman (2017). Melatonin: A pleiotropic molecule that modulates DNA damage response and repair pathways. Journal of Pineal Research.

[8] Yuan Z, Zhang X, Sengupta N, Lane WS, Seto E (2007). SIRT1 regulates the function of the Nijmegen breakage syndrome protein. Mol. Cell.

[9] Cohen HY (2004). Acetylation of the C terminus of Ku70 by CBP and PCAF controls Bax-mediated apoptosis. Mol. Cell.

[10] Yamamori T (2010). SIRT1 deacetylates APE1 and regulates cellular base excision repair. Nucleic Acids Res..

[11] Motta MC (2004). Mammalian SIRT1 represses forkhead transcription factors. Cell.

[12] Singh CK (2014). Novel downstream molecular targets of SIRT1 in melanoma: a quantitative proteomics approach. Oncotarget.

[13] Stunkel W (2007). Function of the SIRT1 protein deacetylase in cancer. Biotechnol. J..

[14] Lee H (2011). Expression of DBC1 and SIRT1 is associated with poor prognosis for breast carcinoma. Hum. Pathol..

[15] Fraga MF, Esteller M (2007). Epigenetics and aging: the targets and the marks. Trends. Genet..

[16] Wang S (2009). JWA regulates XRCC1 and functions as a novel base excision repair protein in oxidative-stress-induced DNA single-strand breaks. Nucleic Acids Res..

[17] Ci Y (2018). SCF(beta-TRCP) E3 ubiquitin ligase targets the tumor suppressor ZNRF3 for ubiquitination and degradation. Protein Cell.

[18] Duan S (2011). mTOR generates an auto-amplification loop by triggering the betaTrCP and CK1alpha-dependent degradation of DEPTOR. Mol. cell.

[19] Xu W (2018). KDM5B demethylates H3K4 to recruit XRCC1 and promote chemoresistance. Int. J. Biol. Sci..

[20] Feng L (2017). Tamoxifen activates Nrf2-dependent SQSTM1 transcription to promote endometrial hyperplasia. Theranostics.

[21] Luo L, King NP, Yeo JC, Jones A, Stow JL (2014). Single-step protease cleavage elution for identification of protein-protein interactions from GST pull-down and mass spectrometry. Proteomics.

[22] Cai J (2013). miR-205 targets PTEN and PHLPP2 to augment AKT signaling and drive malignant phenotypes in non-small cell lung cancer. Cancer Res..

[23] Wang C (2006). Interactions between E2F1 and SirT1 regulate apoptotic response to DNA damage. Nat. Cell Biol..

[24] Jung-Hynes B, Nihal M, Zhong W, Ahmad N (2009). Role of sirtuin histone deacetylase SIRT1 in prostate cancer. A target for prostate cancer management via its inhibition?. J. Biol. Chem..

[25] Shuang T, Wang M, Zhou Y, Shi C (2015). Over-expression of Sirt1 contributes to chemoresistance and indicates poor prognosis in serous epithelial ovarian cancer (EOC). Med. Oncol..

[26] Ma L (2014). SIRT1 and SIRT2 inhibition impairs pediatric soft tissue sarcoma growth. Cell Death Dis..

[27] Nihal M, Ahmad N, Wood GS (2014). SIRT1 is upregulated in cutaneous T-cell lymphoma, and its inhibition induces growth arrest and apoptosis. Cell cycle.

[28] Magni M (2018). TSPYL2 is a novel regulator of SIRT1 and p300 activity in response to DNA damage. Cell Death Differ.

[29] He X, Maimaiti M, Jiao Y, Meng X, Li H (2018). Sinomenine induces G1-phase cell cycle arrest and apoptosis in malignant glioma cells via downregulation of sirtuin 1 and induction of p53 acetylation. Technol. Cancer Res. Treat..

[30] Wei L (2013). Damage response of XRCC1 at sites of DNA single strand breaks is regulated by phosphorylation and ubiquitylation after degradation of poly(ADP-ribose). J. Cell Sci..

[31] Xu W (2014). JWA reverses cisplatin resistance via the CK2-XRCC1 pathway in human gastric cancer cells. Cell Death Dis..

[32] Zhou Q (2019). Inhibiting neddylation modification alters mitochondrial morphology and reprograms energy metabolism in cancer cells. JCI Insight.

[33] Nguyen TV (2016). Glutamine triggers acetylation-dependent degradation of glutamine synthetase via the thalidomide receptor Cereblon. Mol. Cell.

[34] Koirala S, Potts PR (2016). An acetyldegron triggers CRBN to take down the “Q”. Mol. Cell.

