# Supplementary material for: SIRT1 deacetylated and stabilized XRCC1 to promote chemoresistance in lung cancer
Source: Cell Death Dis. 2019 May 1;10(5):363. doi: 10.1038/s41419-019-1592-3 (PMC6494911; doi:10.1038/s41419-019-1592-3)
Supplement: Supplementary file 1 — Supplementary Figures and Tables [file 41419_2019_1592_MOESM1_ESM.pdf]

Supplementary Figures and Figure legends

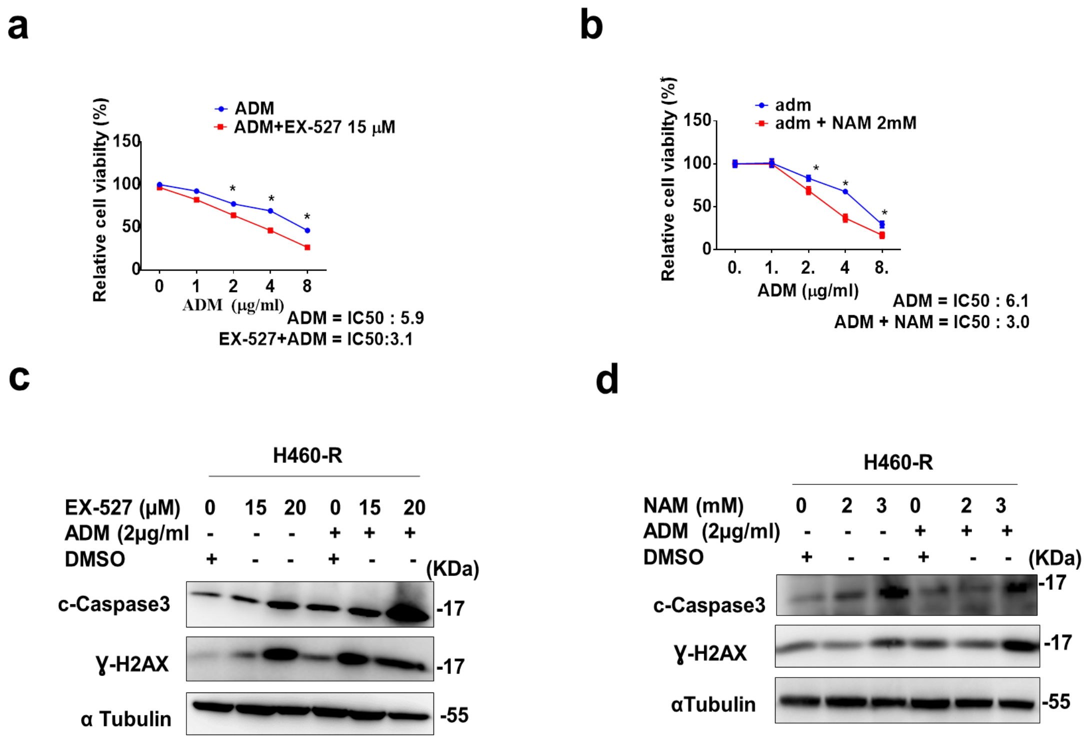

Supplementary Figure 1. Chemical inhibitors of SIRT1 impair chemoresistance. **(**a, b) ADM induced viability inhibition of H460-R with or without EX-527 and NAM treatment (48 Hrs.) were measured by MTS assay. (c, d) c-Caspase3 and ɣ-H2AX levels in H460-R with or without EX-527 and NAM treatment were analyzed by Western blotting with anti- ɣ-H2AX and c-Caspase3 antibody (48 Hrs.).

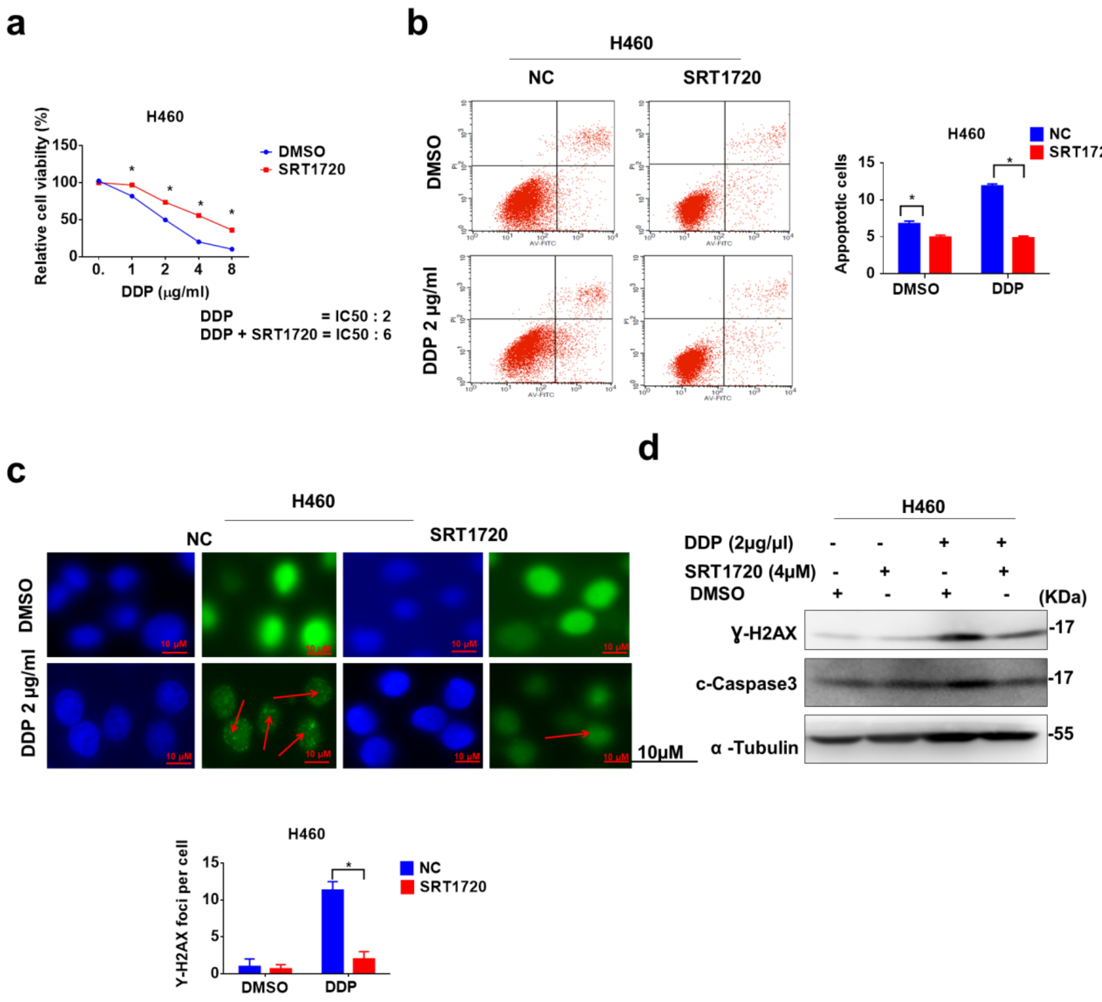

Supplementary Figure 2. Activator of SIRT1 induces chemoresistance to H460 cells. (a) Cell viability of H460 cells were measured by MTS assay after treatment with SRT1720 and cisplatin. (b, c) SIRT1 activator SRT1720 were reduced apoptosis and DNA damage Ɣ-H2AX levels in H460 cells with and without cisplatin treatment. (d) Ɣ-H2AX and c-caspase3 antibody were analyzed by Western blot.

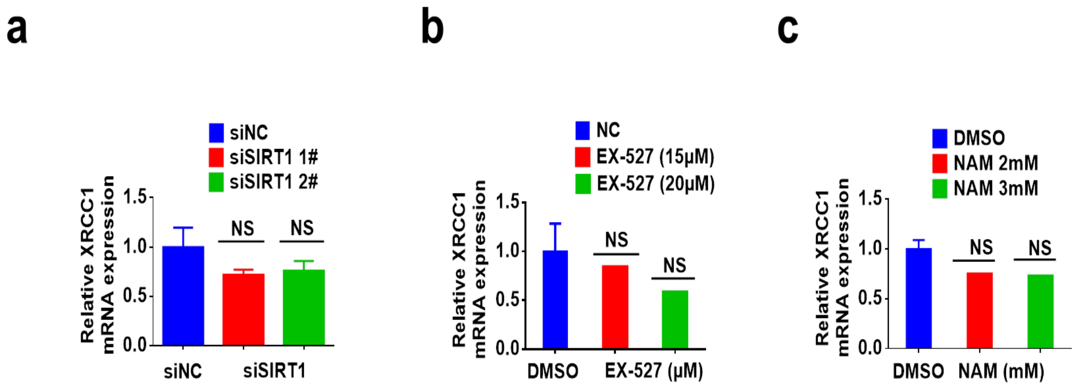

Supplementary Figure 3. XRCC1 mRNA expression. (a-c) XRCC1 mRNA expression in H460-R cells were determined by real time q-PCR (qRT-PCR) after treatment with SIRT1 siRNAs and chemical inhibitors.

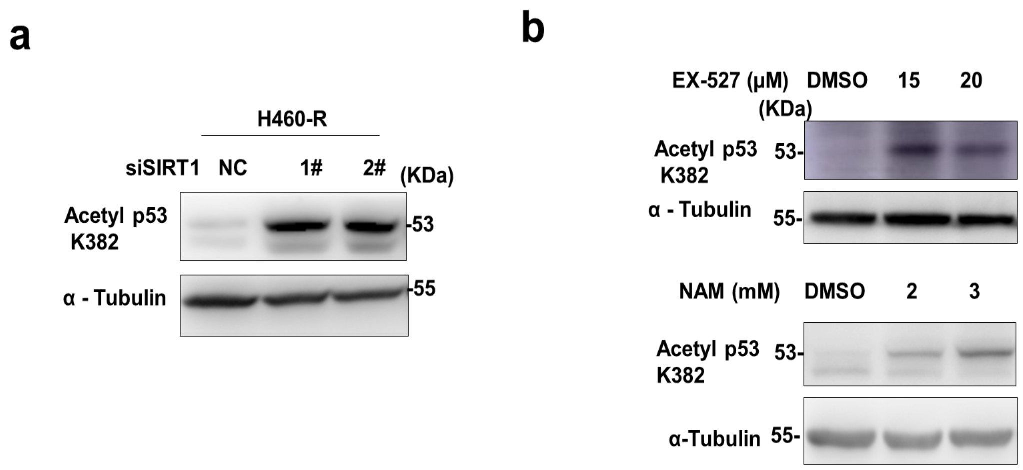

Supplementary Figure 4. SIRT1 inhibition promotes p53 acetylation**.** (a, b) Acetylation of p53 K382 site in H460-R cells after treatment with SIRT1 siRNAs and inhibitors were detected by Western blotting with anti-p53 Acetyl antibody analysis.

Supplementary Table 1. Sequence of siRNAs.

siRNA	Sequence
SIRT1 siRNA 1#	5'-GGAAAUAUAUCCUGGACAATT-3'
SIRT1 siRNA 2#	5'-GCAACUAUACCCAGAACAUTT-3'
β-TrCP siRNA 1#	5-AAGTGGAATTTGTGGAACATC-3'

Supplementary Table 2. The list of primers sequence.

Primer	Sequence
mRNA (XRCC1-F)	5-ACTGCTGGAACCTGGCCCTGC-3'
mRNA (XRCC1-R)	5-GCAAACCCCGAGGAGAAGGCA-3'
WT (XRCC1-F)	5-ATGCCGGAGATCCGC-3'
WT (XRCC1-R)	5-TCAGGCTTGCGGCAC-3'
K 260 to R (XRCC1-F)	5'-CTCTAGGGCCCTATCTCGCAGCTCGGAGCG-3'
K 260 to R (XRCC1-R)	5'-CGCTCCGAGCTGCGAGATAGGGCCCTAGAG-3'
K298 to R (XRCC1-F)	5'-CCTCGGGGTCTGCCTGTCACTGCCCCC-3'
K298 to R (XRCC1-R)	5'-GGGGGCAGTGACAGGCAGACCCCGAGG-3'
K431 to R (XRCC1-F)	5'-CTGGGGTTGCCTCTGAGGAAGCTTGGGGGC-3'

K431 to R (XRCC1-R)	5'-GCCCCCAAGCTTCCTCAGAGGCAACCCCAG-3'
GST-S1 (XRCC1-F)	5'-GGAATTCATGCCGGAGATCCGC-3'
GST-S1 (XRCC1-R)	5'-ATAAGAATGCGGCCGCCTTTGTCTGGGGGGCTATG-3'
GST-S2 (XRCC1-F)	5'-GGAATTCATGCCGGAGATCCGC-3'
GST-S2 (XRCC1-R)	5'-ATAAGAATGCGGCCGCCAGCTCCTCTGGGCCAG-3'
GST-S3 (XRCC1-F)	5'-GGAATTCATGCCGGAGATCCGC-3'
GST-S3 (XRCC1-R)	5'-ATAAGAATGCGGCCGCCTCATCCTCCTCACTGCTGG-3'
GST-S4 (XRCC1-F)	5'-GGAATTCATGCCGGAGATCCGC-3'
GST-S4 (XRCC1-R)	5'-ATAAGAATGCGGCCGCTCAGGCTTGCGGCAC-3'
